# Instability Severity Index Score Does Not Predict the Risk of Shoulder Dislocation after a First Episode Treated Conservatively

**DOI:** 10.3390/ijerph182212026

**Published:** 2021-11-16

**Authors:** Umile Giuseppe Longo, Rocco Papalia, Gianluca Ciapini, Sergio De Salvatore, Carlo Casciaro, Elisa Ferrari, Fabio Cosseddu, Michele Novi, Ilaria Piergentili, Paolo Parchi, Michelangelo Scaglione, Vincenzo Denaro

**Affiliations:** 1 Department of Orthopaedic and Trauma Surgery, Campus Bio-Medico University, 00128 Rome, Italy; r.papalia@unicampus.it (R.P.); s.desalvatore@unicampus.it (S.D.S.); c.casciaro@unicampus.it (C.C.); ilaria.piergentili94@gmail.com (I.P.); denaro@unicampus.it (V.D.); 2 1st Orthopedic Division, Department of Translational Research and New Technologies in Medicine and Surgery, University of Pisa, 56126 Pisa, Italy; gianluca.ciapini@unipi.it (G.C.); paolo.parchi@unipi.it (P.P.); 3 Department of Orthopedic and Traumatology, University of Pisa, 56126 Pisa, Italy; elisa.ferrari@unipi.it (E.F.); fabio.cosseddu@unipi.it (F.C.); michele.novi@unipi.it (M.N.); michelangelo.scaglione@unipi.it (M.S.)

**Keywords:** shoulder instability, Instability Severity Index, shoulder dislocation, ISIS, conservative, surgery, predictivity, recurrent dislocation, risk factor

## Abstract

The first purpose of this study was to verify the association between Instability Severity Index Score (ISIS) and Recurrent Shoulder Dislocation (RSD) after a first episode treated conservatively. The second aim is to identify the risk factors associated with RSD after a primary acute shoulder anterior dislocation treated conservatively. A total of 111 patients with first traumatic anterior shoulder dislocation treated at a single trauma centre between January 2014 and March 2016 were enrolled. The main predictive variables of risk factors and the ISIS score were calculated. Among the 85 patients included, 26 cases of RSD were observed (30.6%). Considering the whole population, no significant association between ISIS and RSD were reported. Regarding other risk factors, high-risk working activities and rotator cuff injury had a significantly higher RSD risk. Sex, dominant limb, familiar history, hyperlaxity, contact or overhead sports, competitive sport, post-reduction physiokinesitherapy, return to sports activity time, Hill-Sachs lesion, bony Bankart lesion and great tuberosity fracture did not seem to influence the risk of RSD. No correlation between ISIS score and RSD in patients treated conservatively after a first episode of shoulder dislocation were reported. The only risk factors with a significant association to RSD were high-risk working activities and rotator cuff injury.

## 1. Introduction

The shoulder is the most frequently dislocated joint in the human body. A complete pop out of the humeral head from the glenoid socket is defined as dislocation [1]. The inability of bone and ligamentous structures of the shoulder joint to maintain the humeral head in the glenoid fossa, could cause a condition known as “shoulder instability” [2].

The incidence of shoulder dislocation is estimated between 11 and 51 cases per 100,000 individuals per year [3,4,5], but it is highly variable, as it depends on the population studied. The rate is significantly higher when considering an active population such as the military or athletes [4,5]. Anterior traumatic dislocation is the most frequent type, representing about 96% of all glenohumeral dislocations [6]. Immediate joint reduction and immobilization, followed by rehabilitation, is the most common conservative treatment for primary anterior dislocations. Surgical treatment, however, is generally used in case of recurrence or chronic instability [7]. In the literature, Recurrent Shoulder Dislocation (RSD) following a first traumatic episode ranges from 26% [8] to 92% [9,10].

Young age, male sex [11], and contact sports [12] seem to increase the risk of instability after a primary anterior dislocation, but other studies reported discordant findings [13]. Other risk factors are the type of trauma [14], constitutional hyperlaxity [15], and the presence of a Hill-Sachs lesion on radiography [16].

The Instability Severity Index Score (ISIS) score was created to guide the surgeon’s decision identifying patients with a high risk of RSD after an arthroscopic Bankart surgery [17]. The risk factors considered by this score are age at surgery, glenoid loss of contour on AP radiograph, Hill-Sachs lesion on external rotation anteroposterior (AP) radiograph, degree of sports participation, type of sport and shoulder hyperlaxity [17]. Although ISIS and other scores have tried to assess the risk of RSD after surgery, limited findings are reported on risk factors for RSD after a first episode of dislocation treated conservatively [13,14].

The first purpose of this study is to verify the association between ISIS and RSD after a first episode treated conservatively. The second aim is to identify the risk factors associated with RSD after a primary acute shoulder anterior dislocation treated conservatively.

## 2. Materials and Methods

### 2.1. Participants and Setting

**Figure 1:**
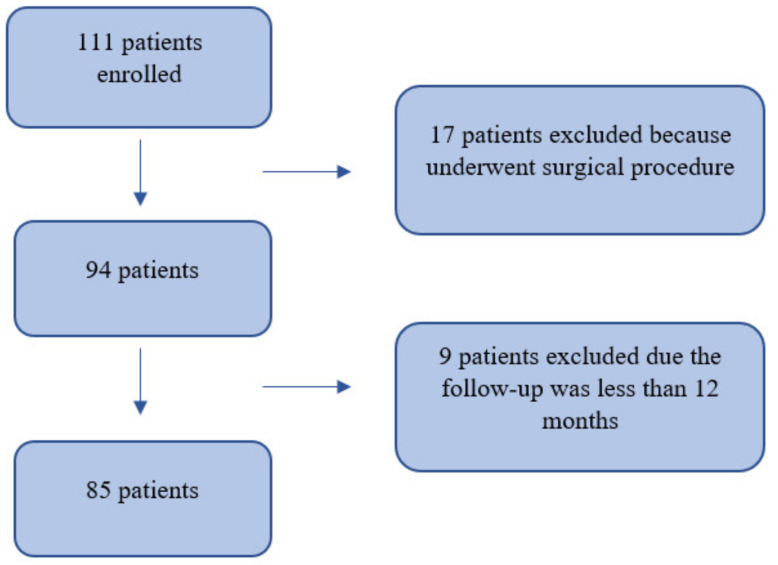
Flow chart.

Between January 2014 and March 2016, 111 patients with first traumatic anterior shoulder dislocation treated at a single trauma centre were enrolled. A total of 17 patients were excluded because they underwent a surgical procedure, while 9 patients were excluded due having a follow-up less than 12 months. Therefore, 85 patients were included in this study (Figure 1). The inclusion and exclusion criteria are summarized in Table 1. RSD is defined according to Hobby et al. as “radiological documentation of further dislocation, recurrence of dislocation by re-injury requiring manual reduction, recurrence of symptoms of the shoulder “popping out” or “slipping out” in a position of abduction and external rotation suggestive of subluxation, or symptomatic subluxation or instability preventing the return to full activity [18,19].

All patients were treated with immobilization using a sling with the arm held in external rotation, neutral flexion, and 15° of abduction for four weeks [20]. All patients were recommended the same physiokinesitherapy; after four weeks of immobilization, active-assisted shoulder range of motion exercises, avoiding elevation or abduction of >90° or external rotation of >30°; after twelve weeks, free range of motion; isometric rotator cuff strengthening exercises at six weeks, and the isotonic exercises at twelve weeks. According to the selection criteria, 94 patients were included in the study. Two orthopaedic surgeons interviewed the patients. Two experienced radiologists viewed X-ray and MRI examinations of the affected shoulder of inclusion patients. In case of controversy, a discussion between the radiologist and the senior orthopaedic surgeon solved the conflicts. The Institutional Review Board (IRB) approved the study before data collection. The mean follow-up time was considered as time elapsed since the injury and the moment of the interview.

### 2.2. Data Collection

The main predictive variables, including age, gender, affected side, dominant arm, family history of dislocations and hyperlaxity (hyperlaxity is a physiological condition characterized with increased range of motion of various joints of an individual [21]), were recorded during a clinical examination. Patients were divided into two groups accordingly to their working activity, high-risk jobs, and non-high-risk jobs (Table 2). The sports activity practised, and the level (competitive or non-competitive) was recorded and divided into two main types, sports that require contact or overhead force and other sports. The eventual post-reduction physiokinesitherapy performed and the time to return to sports (expressed in months) were reported. Associated injuries such as Hill-Sachs, Bony Bankart, rotator cuff injury, or greater tuberosity fracture were assessed on X-rays and MRI. According to the University of California Los Angeles Shoulder Scale (UCLA) [22] all patients were evaluated for pain and function. Moreover, The Short Shoulder Test (SST) [23] and the Shoulder Pain and Disability Index (SPADI) [24] functional scales were assessed. The ISIS [17] was calculated by the senior surgeon after the first episode of shoulder dislocation. In Table 2, all variables included in this study are described.

### 2.3. Statistical Analysis

A priori power analysis was conducted to assess the minimum number of patients required for the study. Assuming a recurred dislocation after conservative treatment of 37.5% as in literature [7], an effect size of 0.2, using α = 0.05, power of 80% and a two-tailed test, a sample size of 52 patients was required.

Data were collected in Microsoft Excel. To assess data normality the Shapiro–Wilk test was used. To determine the statistical differences in the months of follow-up between RDS and not RDS, an independent t-test was used. A binary logistic regression model was used to study the correlations between risk factors, ISIS and the presence of RSD. Demographic data were calculated as descriptive variables using frequencies, means and standard deviation (SD). The statistical analysis was conducted using IBM SPSS (v 26) software (IBM, Armonk, NY, USA). A *p*-value ≤ 0.05 was considered as significative.

## 3. Results

A cohort of 85 patients with a first episode of anterior shoulder dislocation was enrolled for this study (67 males and 18 females), with a mean age of 40 ± 14.7 (range 18–65 years old). Of these, 60 patients were injured at the right shoulder instead of 25 at the left shoulder. The first episode of shoulder dislocation occurred in 57 cases in the dominant limb (55 right shoulders; 2 left shoulders). The mean follow-up (intended as time elapsed since the first episode) was 48.1 ± 25.7 months (range 12–156 months). Among the 85 patients included, 26 cases of RSD were observed (30.6%). All descriptive statistical analyses were presented in Table 2.

No statistically significant association between ISIS and RSD were reported (*p* = 0.4, Table 3).

Regarding other risk factors, patients who sustained high-risk working activities had an Odds Ratio (OR) of 4.3 (95%CI = [1.2, 15.5]; *p* = 0.02) compared to other patients. In patients with a rotator cuff injury the OR was 10.8 (95%CI = [3.1, 37.9]; *p* < 0.001) compared to other patients.

Concerning other variables (sex, dominant limb, familiar history, hyperlaxity, contact or overhead sports, competitive sport, post-reduction physiokinesitherapy, return to sports activity time, Hill-Sachs lesion, Bony Bankart lesion, greater tuberosity fracture, UCLA Score, SPADI Score, SST Score) no significant association were found. Only 52/85 (61.2%) of the patients undertook physiokinesitherapy after the first episode of dislocation.

A statistically significant difference in average months of follow-up between the group with and without recurrence of dislocation was found (*p* = 0.04). The mean months of follow-up in RDS group was 59 ± 33.8, while in the not RDS group 43.4 ± 19.7 months.

## 4. Discussion

The main findings of this study are that among the 85 patients analysed, RSD after a first episode of shoulder dislocation treated conservatively were reported in 30.6% of cases. ISIS was analysed, and no significant association with the risk of RSD was reported.

### 4.1. Predictivity of ISIS for RSD

Balg and Boileau [17] developed the ISIS score to evaluate the preoperative risk of recurrent instability after an arthroscopic repair, based on six risk factors. They found a 70% risk of RSD after surgery in case of ISIS > 6 points [17]. Phadnis et al. [25] questioned the real efficacy of ISIS, emphasizing the potential advantages and disadvantages. They concluded that even with the lower value of ISIS (score > 4) there is a 70% risk of RSD, in opposition to the results of Balg [17]. There is a lack of literature concerning the use of ISIS in patients treated conservatively. In this study, a possible association between ISIS and RSD after a first episode of dislocation treated conservatively was evaluated. Otherwise, no statically significant association between ISIS (assessed after the first episode of dislocation) and further RSD were founded (neither stratifying the population by age, as reported in Table 3). Other scores such as UCLA, SPADI and SST were analysed, but no significant association with RSD was found. These scores assess shoulder pain and functionality; instead, ISIS investigate the risk of RSD. Therefore, the lack of correlation between UCLA, SPADI, SST and ISIS could be explained by the different aims of the scores. The risk factors that constitute the ISIS were individually analysed.

### 4.2. Risk Factors of RSD after a First Episode Treated Conservatively

Different studies [26,27,28] have focused on risk factors for RSD identifying the following variables: age, high-risk working activities, rotator cuff injury, contact or overhead sports, sex, trauma on the dominant limb, familiar history, hyperlaxity [29], competitive sport, post-reduction physiokinesitherapy, return to sports activity time, Hill-Sachs lesion [30], Bony Bankart lesion [31] and great tuberosity fracture. Olds et al. [32] identified 39% of the rate of RSD after one year from the first episode of dislocation. In our study of 85 patients, only 26 (30.6%) experienced RSD. This value is lower than other studies [33], because only patients treated conservatively were included in the analysis. Otherwise, in a systematic review of 2014 [7] the rate of RSD for patients treated conservatively was 37.5%, similar to the results of this study.

The second aim of this paper was to identify risk factors for RSD after a first episode of dislocation treated conservatively. Comparing patients with and without RSD, only two parameters were found to be statistically significant. High-risk working activities showed a significant association with RSD (*p* = 0.02). This could probably be due to the repeated stresses and traumas on the shoulder that increase the risk of recurrence. Moreover, a previous rotator cuff injury seems to have a significant association with RSD (*p* < 0.001).

Surprisingly, sex, dominant limb, familiar history, hyperlaxity, competitive sport, post-reduction physiokinesitherapy, return to sports activity time, Hill-Sachs lesion, bony Bankart lesion and great tuberosity fracture did not have a significant association with the risk of RSD.

### 4.3. Limitations

This study has some limitations. Physiokinesitherapy was prescribed to all the patients with specific indications, but we were not certain how it was performed. Moreover, only 60.6% of patients included in the study performed physiokinesitherapy.

Lastly, the strength of this study is that it was conducted only on patients affected by anterior dislocation, demonstrating the selectivity of the results obtained.

## 5. Conclusions

No correlation between the ISIS score and RSD in patients treated conservatively after a first episode of shoulder dislocation were reported. The only risk factors with a significant association to RSD were high-risk working activities and rotator cuff injury. Sex, dominant limb, familiar history, hyperlaxity, competitive sport, post-reduction physiokinesitherapy, return to sports activity time, Hill-Sachs lesion, bony Bankart lesion and great tuberosity fracture did not seem to influence the risk of RSD. An evaluation of the UCLA, SPADI and SST score was performed, and no significant association was found. Further studies with a higher number of patients are needed to confirm the data obtained.

## Figures and Tables

**Table 1:** Inclusion and exclusion criteria.

Inclusion Criteria	Exclusion Criteria
First traumatic anterior shoulder dislocation treated conservatively	Posterior or multidirectional dislocation
X-ray photography of the affected shoulder	Previous shoulder instability
MRI of the affected shoulder	Atraumatic shoulder dislocation
Age between 18 and 65 years old	Pathologies affecting the acromioclavicular joint
12 months minimum of follow-up	Absence of radiographs and MRI of the affected shoulder
	Fractures (greater tuberosity fractures and bony Bankart lesions except)
	Voluntary dislocations

**Table 2:** Descriptive statistical analyses, frequencies and means of variables included.

Age	Mean age: 40 ± 14.7 (range 18–65 years)
Gender	Males = 67, Females = 18
Affected side	Right = 60, Left = 25
Dominant arm	Yes = 57, No = 28
Family history of dislocations	Yes = 0, No = 85
Hyperlaxity	Yes = 2, No = 83
Working activity	High-risk jobs (paratrooper, bricklayer, military, police, mechanic, jockey, warehouse worker) = 15; Non-high-risk jobs (painter, housewife, pensioner, pharmacist, engineer, waiter, student, driver, fisherman, chef, employee, doctor, computer scientist, trader, hairdresser) = 70
Sports activity practised	Sports that require contact or overhead forced (basket, soccer, martial arts, weightlifting, bodybuilding, rugby, military, tennis, motorbike, swimming and volleyball) = 39; Other sports (fitness exercise, cycling, horseback riding, jogging, skiing, immersion, athletics) = 46
Level of sport	No sport = 29, Non-Competitive = 46, Competitive = 10
Post-reduction physiokinesitherapy	Yes = 52, No = 33
Time to return to sports	From 1 to 8 months
Hill-Sachs	Yes = 14, No = 71
Bony Bankart	Yes = 5, No = 80
Rotator cuff injury	Yes = 17, No = 68
Greater tuberosity fracture	Yes = 3, No = 82
UCLA Score	Mean score: 17.4 ± 3.5
SST Score	Mean score: 9.2 ± 18.1
SPADI Score	Mean score: 84.7 ± 26.4
ISIS Score	Score 0–2 = 65, score 3–6 = 19, score > 6 = 1

**Table 3:** Variables associated with the presence of RSD, analysed using binary regression logistic model.

Variables	*p*-Value
ISIS	0.441
High-risk working activities	0.024 *
Rotator cuff injury	<0.001 *
Contact or overhead sports	0.127
Sex	0.389
Age	0.435
Dominant limb	0.777
Familiar history	N/A
Hyperlaxity	0.577
Competitive sport	0.383
Post-reduction physiokinesitherapy	0.180
Return to sports activity time	0.977
Hill-Sachs lesion	0.252
Bony_Bankart lesion	0.999
Great tuberosity fracture	0.999
UCLA Score	0.369
SPADI Score	0.879
SST Score	0.806

* Statistically significant, NA = Not Applicable.

## Data Availability

The data presented in this study are available on request from the corresponding author. The data are not publicly available due to the privacy.
